# Long-term Survival of Midurethral Mesh Slings for Women with Stress Urinary Incontinence

**DOI:** 10.1016/j.euros.2025.11.012

**Published:** 2025-12-04

**Authors:** Margaux Laude, Imad Bentellis, Roxane Fabre, Matthieu Durand, Brannwel Tibi, Céline Chauleur, Benoit Peyronnet, Laurent Bailly

**Affiliations:** a Department of Gynecology and Obstetrics, University Hospital Saint-Etienne, France; b Department of Urology, Hospital Pasteur 2, University Hospital of Nice, France; c Public Health Department, University Hospital Nice, Université Côte d’Azur, Nice, France; d Clinical Research Unit of the Côte d’Azur, Université Côte d’Azur, Nice, France; e Department of Urology, University of Rennes, France

**Keywords:** Stress urinary incontinence, Midurethral sling, Removal, Reinsertion, Surgery, Risk factors

## Abstract

**Background and objective:**

Midurethral sling (MUS) use for stress urinary incontinence (SUI) has declined because of controversies. However, there is little evidence on the long-term prevalence of MUS reoperation. The aim of this study was to examine long-term removal and reoperation rates among women with MUS.

**Methods:**

This national retrospective cohort study included all female patients aged ≥18 yr with a first MUS insertion for SUI in all French public hospitals and private practices between January 1, 2013, and September 30, 2022. Patients were followed until January 1, 2023. The primary outcome was the MUS removal rate. Secondary outcomes were new MUS insertion and any other surgery for SUI. Cox regression models were used to assess potential factors associated with MUS removal.

**Key findings and limitations:**

The study included 217 326 women with a first MUS insertion. There were 5851 MUS removals and 9521 new MUS insertions. Most removals occurred within the first year (58%). The MUS removal rate was 2.7% at 10 yr. There were fewer removals after transoboturator insertion than after retropubic insertion. Alcohol consumption, smoking, and obesity were associated with MUS reoperation. At 10 yr, the rate of new MUS insertions was 4.5% and the rate of any other surgeries was 2.8%.

**Conclusions and clinical implications:**

These findings may help in counseling patients on decision-making for SUI treatment.

**Patient summary:**

One treatment option for stress urinary incontinence (SUI) in women is insertion of a mesh sling. We looked at the rates of sling removal and reoperation among women treated for SUI in the French population. We found that after 10 years, only 2.7% of cases needed sling removal, 4.5% needed a new sling inserted without removal of the previous sling, and 2.8% needed another type of surgery. These results will help patients in choosing the correct treatment for SUI.

## Take Home Message

We conducted a retrospective nationwide French cohort study using data for 217 326 women who had a midurethral sling inserted for stress urinary incontinence over a study period of 10 yr. The rate of midurethral sling removal estimated at 10 yr was 2.7%.

## 1 Introduction

Urinary incontinence, defined as unintentional and uncontrolled release of urine, is a highly prevalent condition among women [1]. The most common type is stress urinary incontinence (SUI), defined as involuntary urine leakage not preceded by an urge to void that occurs during physical activities. SUI affects approximately one in three women and has a severe impact on patients’ quality of life [2], [3], [4].

When pelvic-floor muscle training fails to improve SUI, female patients are offered surgical options. Since its description in the mid 1990s, the midurethral sling (MUS) has become the most widely used surgical treatment for female SUI. This approach entails insertion of a polypropylene mesh between the vaginal and urethral wall using a small incision via a retropubic (RP) or transoboturator (TO) route [5], [6].

Despite the effectiveness of this option, MUS use has declined recently because of growing patient concerns about potential complications such as chronic pain, MUS exposure in the vagina, and extrusion in the urinary tract [2], [6]. Some countries, such as the UK and Australia, have banned the use of all vaginal mesh products for SUI and pelvic organ prolapse [7], [8]. In the USA, vaginal mesh is very strictly regulated [9]. However, the prevalence of these debilitating complications is not clear, as there are few population-based studies assessing long-term MUS outcomes [10], [11], [12].

The aim of our study was to examine long-term MUS removal and reoperation rates among women and to explore factors associated with reoperation.

## 2 Patients and methods

The study protocol was approved by the French National Health Insurance Information System (Système National des Données de Santé, SNDS) and the French National Health Data Institute (Institut National des Données de Santé) [13].

The study poses no ethical problem, no stigmatization of a specific group and does not go against morality. The SNDS data were used in accordance with current regulations and are anonymous and subject to strict regulations and quality standards.

### 2.1 Study design

We conducted a national retrospective epidemiological study using the French claims database (Programme de Médicalisation des Systèmes d’Information; PMSI). This database contains records for all inpatient admissions in French public and private hospital, including demographic (age, comorbidities), admission (date of admission and discharge), and clinical data. Diagnostic information is coded using the International Statistical Classification of Diseases, tenth revision [14]. Surgical interventions were analyzed using French Common Classification of Medical Acts (CCAM) codes [15]. It has been demonstrated that the accuracy of PMSI data is sufficiently robust to support their use for research [16].

We included all patients aged ≥18 yr who received a first MUS insertion for SUI in a public or private health care institution in France between January 1, 2013 and September 30, 2022.

SUI is defined by the International Statistical Classification of Diseases and Related Health Problems, Tenth Revision, codes N393 (Stress Urinary Incontinence) and R32 (Urinary Incontinence, unspecified). All N394 codes (Other specified forms of urinary incontinence) were excluded. Synthetic MUS insertions were defined by the CCAM codes JDDB005 (Bladder support by infra-urethral synthetic sling, transvaginally and transobturator) and JDDB007 (Bladder support with infra-urethral synthetic sling, transvaginally and transretropubically, with endoscopic control).

The procedure was considered a first MUS insertion if there was no record of an SUI surgery code before the insertion code during the study period.

Participants were followed from the date of the initial procedure until the date of death or the end of the follow-up period on January 1, 2023.

### 2.2 Outcomes

The primary outcome was MUS removal, defined as partial (CCAM codes JRGA002, JRGC001) or total (JRGA001, JRGA003) removal or MUS section (JRPA001) after initial insertion. Further code definitions can be found in Supplementary Table 1.

The secondary outcomes were new MUS insertion without removal, defined as a subsequent MUS insertion (CCAM codes in Supplementary Table 1), and other subsequent SUI surgery excluding MUS removal and reinsertion.

### 2.3 Patient factors

The following data for each patient were extracted from the PMSI database: age at the time of MUS insertion, comorbidities (Supplementary Table 2), and insertion route (RP vs TO).

Data on race and ethnicity could not be collected, because this is considered unethical in France.

### 2.4 Statistical analyses

Cumulative incidence functions were used to evaluate the risk of MUS removal and reoperation as a function of time from the initial procedure to first removal or reoperation, with death as a competing event and patients reaching the end of the follow-up period as a censoring event.

All statistical analyzes were carried out using SAS (SAS Institute, Cary, NC, USA; https://www.sas.com) and R (R Foundation for Statistical Computing, Vienna, Austria; https://www.r-project.org/) software. Descriptive, univariate, and multivariate analyzes were performed. All statistical tests are two-sided and considered significant at the 5% level.

Patient demographic and clinical characteristics are reported as the number and proportion for categorical variables, and as the median and interquartile range (IQR) for continuous variables.

## 3 Results

**Fig. 1:**
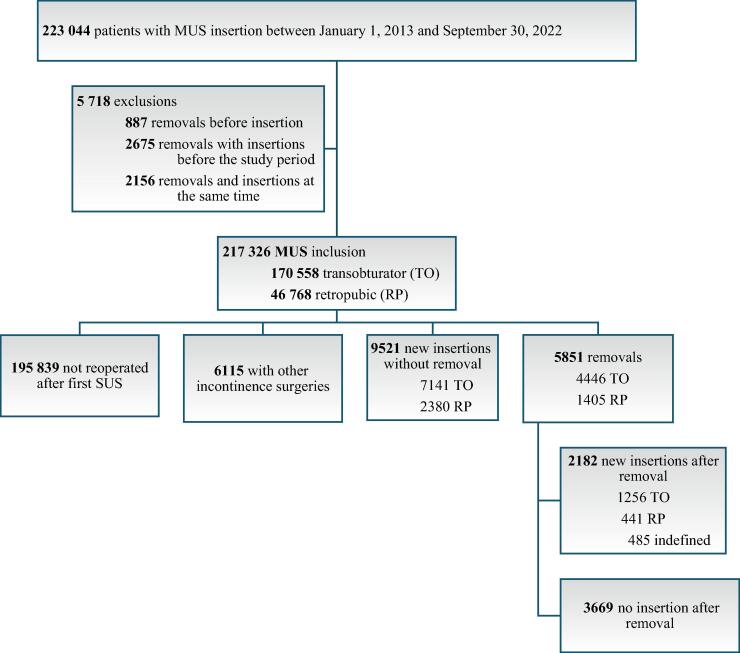
Study population of women aged ≥18 yr who had a first midurethral sling (MUS) inserted in a French hospital between January 1, 2013 and September 30, 2022.

We identified 223 044 patients with a first MUS insertion between January 2013 and September 2022. Of these, 5718 patients were excluded because they did not meet our inclusion criteria (Fig. 1).

The total study population comprised 217 326 women with a first MUS insertion, including 46 768 (21.5%) in the RP group and 170 558 (78.5%) in the TO group (Table 1). Median follow-up was 2.4 yr (IQR 1–5.2). The median age was 56 yr (IQR 47–68), and 24% of the women had one or more comorbidities.

**Table 1:** Sling removal following initial insertion

Parameter	Patients, *n* (%)	Sling removal, *n* (%)				HR at 10 yr (95% CI)	*p* value
		6 mo	1 yr	5 yr	9 yr		
Cumulative incidence	217 326 (100)	2396 (1.1)	3389 (1.6)	5399 (2.5)	5851 (2.7)		
Age at initial surgery							
18–39 yr	16 256 (7.5)	259 (1.6)	360 (2.2)	540 (3.3)	571 (3.5)		
40–49 yr	56 989 (26.2)	700 (1.2)	960 (1.7)	1500 (2.6)	1655 (2.9)		
50–59 yr	52 330 (24.1)	567 (1.1)	800 (1.5)	1329 (2.5)	1457 (2.8)	1 (reference)	
60–69 yr	46 787 (21.5)	421 (0.9)	608 (1.3)	1026 (2.2)	1119 (2.4)	0.998 (0.996–1.000)	**0.0210**
≥70 yr	44 964 (20.7)	449 (1.0)	661 (1.5)	1004 (2.2)	1049 (2.4)		
Surgery setting							
Outpatient	102 042 (47.0)	1020 (1.0)	1428 (1.4)	2245 (2.2)	2449 (2.4)	0.904 (0.857–0.953)	**<0.01**
Inpatient	115 284 (53.0)	1383 (1.2)	1960 (1.7)	3113 (2.7)	3458 (3.0)	1 (reference)	
Insertion route							
Transobturator	170 558 (78.5)	1705 (1.0)	2469 (1.5)	4097 (2.4)	4446 (2.6)	0.899 (0.846–0.955)	**<0.01**
Retropubic	46 768 (21.5)	701 (1.5)	920 (2.0)	1302 (2.8)	1405 (3.0)	1 (reference)	
Comorbidities							
Obesity	33 023 (15.2)	396 (1.2)	594 (1.8)	991 (3.0)	1090 (3.3)	1.071 (1.002–1.145)	**0.0429**
No obesity	184 303 (84.8)	2027 (1.1)	2765 (1.5)	4423 (2.4)	4792 (2.6)	1 (reference)	
AC/S	12 648 (5.8)	190 (1.5)	266 (2.1)	455 (3.6)	506 (4.0)	1.175 (1.071–1.289)	**<0.01**
No AC/S	204 678 (94.2)	2251 (1.1)	3070 (1.5)	4912 (2.4)	5322 (2.6)	1 (reference)	

AC/S = alcohol consumption or smoking; CI = confidence interval; HR = hazard ratio.

MUS removal occurred in 5851 patients, new MUS insertion without removal in 9521 patients, and other SUI surgeries after MUS insertion in 6115 patients. A total of 195 839 women had no repeat surgery.

### 3.1 MUS removal

**Fig. 2:**
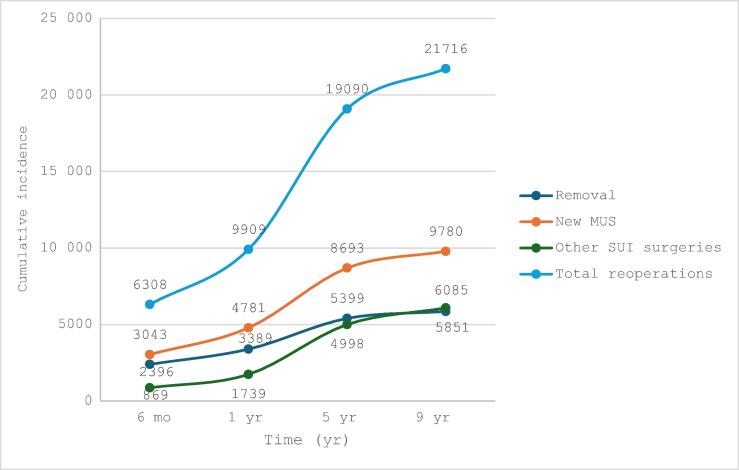

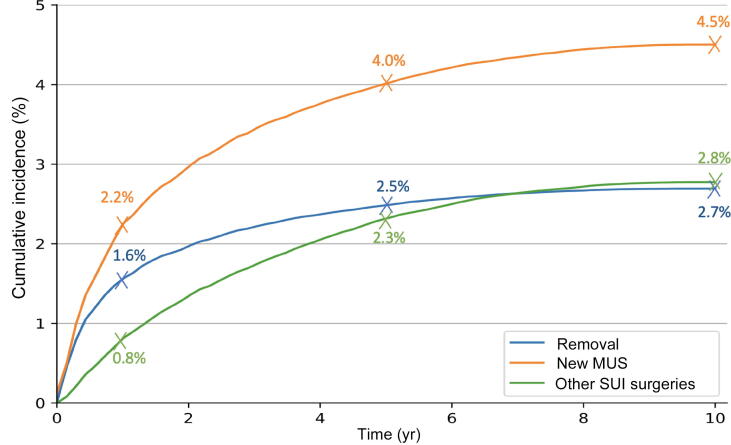
(A) Rates of MUS removal, MUS reinsertion without removal, and other subsequent surgical procedures for stress urinary incontinence (SUI) according to time after initial mesh insertion in the study population (217 326 women): (A) cumulative number and (B) cumulative incidence. MUS = midurethral sling.

Data for MUS removal at 1 yr, 5 yr, and 10 yr are shown in Table 1 and Figure 2. Most MUS removals happened within the first year (3389 patients).

**Fig. 3:**
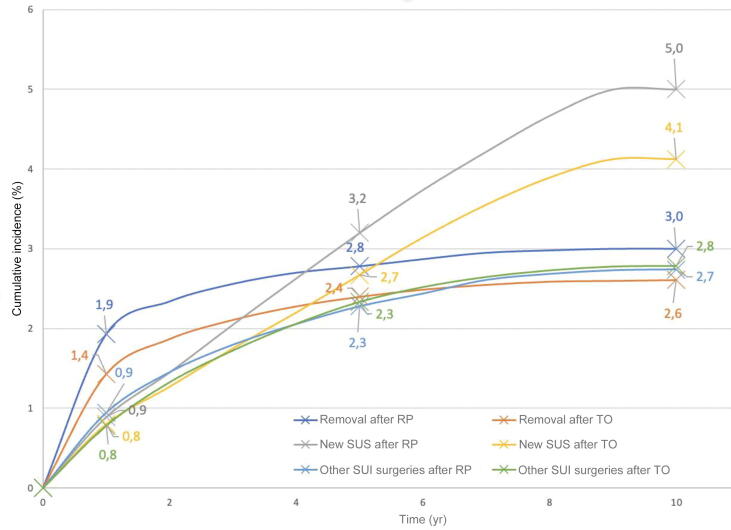
Cumulative incidence rates for MUS removal, MUS reinsertion without removal, and other subsequent surgical procedures for stress urinary incontinence (SUI) according to time after initial insertion in the study population (217 326 women) by insertion route. MUS = midurethral sling; RP = retropubic; TO = transobturator.

The risk of removal at all time points was lower in the TO group than in the RP group (adjusted hazard ratio [HR] 0.899, 95% confidence interval [CI] 0.846–0.953; Fig. 3). The risk of MUS removal decreased with age (adjusted HR 0.998, 95% CI 0.996–1.000; Table 1) and was lower for outpatient than for inpatient surgery (adjusted HR 0.904, 95% CI 0.857–0.953).

Among all the comorbidities assessed, alcohol consumption and/or smoking and obesity were the only factors associated with MUS removal, with adjusted HRs of 1.175 (*p* < 0.01) and 1.071 (*p* = 0.04), respectively.

### 3.2 MUS reinsertion without removal

Data for MUS reinsertion at 1 yr, 5 yr, and 10 yr are shown in Table 2 and Figure 2.

**Table 2:** Sling reinsertion without removal following initial insertion

Parameter	Patients, *n* (%)	Reinsertion without removal, *n* (%)				HR at 10 yr (95% CI)	*p* value
		6 mo	1 yr	5 yr	9 yr		
Cumulative incidence	217 326 (100)	2961 (1.4)	4662 (2.1)	8465 (3.9)	9501 (4.4)		
Age at initial surgery							
18–39 yr	16 256 (7.5)	157 (1.0)	230 (1.5)	499 (3.1)	566 (3.5)		
40–49 yr	56 989 (26.2)	553 (1.0)	788 (1.4)	1491 (2.6)	1652 (2.9)		
50–59 yr	52 330 (24.1)	560 (1.1)	860 (1.6)	1549 (3.0)	1759 (3.4)	1 (reference)	
60–69 yr	46 787 (21.5)	731 (1.6)	1156 (2.5)	2037 (4.4)	2269 (4.9)	1.022 (1.021–1.024)	**<0.01**
≥70 yr	44 964 (20.7)	1054 (2.3)	1628 (3.6)	2889 (6.4)	3255 (7.3)		
Surgery setting							
Outpatient	102 042 (47.0)	1295 (1.3)	1992 (2.0)	3287 (3.2)	3685 (3.6)	0.680 (0.623–0.735)	
Inpatient	115 284 (53.0)	1678 (1.5)	2797 (2.4)	5146 (4.5)	5817 (5.0)	1 (reference)	**<0.01**
Insertion route							
Transobturator	170 558 (78.5)	2325 (1.4)	3500 (2.1)	6354 (3.7)	7128 (4.2)	0.770 (0.735–0.806)	**<0.01**
Retropubic	46 768 (21.5)	725 (1.6)	1162 (2.5)	2111 (4.5)	2373 (5.1)	1 (reference)	
Comorbidities							
Obesity	33 023 (15.2)	734 (2.2)	1146 (3.5)	2308 (7.0)	2655 (8.0)	1.434 (1.370–1.500)	**<0.01**
No obesity	184 303 (84.8)	2334 (1.3)	3516 (1.9)	6157 (3.3)	6846 (3.7)	1 (reference)	
AC/S	12 648 (5.8)	304 (2.4)	432 (3.4)	831 (6.6)	937 (7.4)	1.412 (1.319–1.512)	**<0.01**
No AC/S	204 678 (94.2)	2791 (1.4)	4230 (2.1)	7634 (3.7)	8564 (4.2)	1 (reference)	

AC/S = alcohol consumption or smoking; CI = confidence interval; HR = hazard ratio.

The risk of MUS reinsertion without removal was lower for the TO group than for the RP group (adjusted HR 0.770, 95% CI 0.735-0.806, Table 2 and Fig. 3). The risk of reinsertion increased with age (adjusted HR 1.022, 95% CI 1.021–1.024; Table 2) and was higher for inpatient than for outpatient surgery (adjusted HR 1.320, 95% CI 1.265–1.377). Alcohol consumption and/or smoking and obesity were the only factors associated with MUS reinsertion, with adjusted HRs of 1.4 (*p* < 0.01) for both.

### 3.3 Other subsequent SUI surgery

Data for subsequent surgical procedures for SUI (excluding MUS removal or reinsertion) at 1 yr, 5 yr, and 10 yr following MUS insertion are shown in Table 3 and Figure 2. The risk at 10 yr was slightly higher in the TO group than in the RP group (adjusted HR 1.087, 95% CI 1.022–1.156; Table 3). The risk of subsequent SUI surgery decreased with age (adjusted HR 0.975, 95% CI 0.973–0.977) and was lower for outpatient than for inpatient surgery (adjusted HR 0.853, 95% CI 0.810–0.899). Among the comorbidities investigated, only obesity was associated with subsequent SUI surgery (adjusted HR 1.2; *p* < 0.01).

**Table 3:** Other surgical treatment (excluding sling removal or reinsertion) for stress urinary incontinence following mesh sling insertion

Characteristic	Patients, *n* (%)	Other surgical treatment, *n* (%)				HR at 10 yr (95% CI)	*p* value
		6 mo	1 yr	5 yr	9 yr		
Cumulative incidence	217 326 (100)	869	1739	4998	6085		
Age at initial surgery							
18–39 yr	16 256 (7.5)	81	146	504	683		
40–49 yr	56 989 (26.2)	228	513	1824	2223		
50–59 yr	52 330 (24.1)	157	314	994	1151	1 (reference)	
60–69 yr	46 787 (21.5)	140	328	795	936	0.975 (0.973–0.977)	**<0.01**
≥70 yr	44 964 (20.7)	225	405	854	944		
Surgery setting							
Outpatient	102 042	306	714	2143	2551	0.855 (0.811–0.901)	**<0.01**
Inpatient	115 284	461	1038	2767	3459	1 (reference)	
Insertion route							
Transobturator	170 558	682	1364	3923	4776	1.084 (1.018–1.154)	**<0.01**
Retropubic	46 768	234	421	1029	1263	1 (reference)	
Comorbidities							
Obesity	33 023	165	363	1123	1354	1.263 (1.187–1.345)	**<0.01**
No obesity	184 303	737	1290	3870	4608	1 (reference)	
Alcohol consumption/smoking	12 648	76	152	430	506	1.056 (0.962–1.158)	0.2529
No alcohol consumption/smoking	204 678	869	1739	4998	6085	1 (reference)	

CI = confidence interval; HR = hazard ratio.

## 4 Discussion

To the best of our knowledge, this is first population-based study on long-term reoperation rates after MUS insertion for SUI in France. As the PMSI database contains records for all inpatient admissions in French public and private hospitals, 100% of incontinence procedures carried out in France were included. We found relatively low rates of long-term MUS reoperation, consistent with what has been observed in other health care systems. In the most robust study to date, Gurol-Urganci et al [17] included 95 000 patients treated between 2006 and 2015 and found that the risk of MUS removal was 3.3% at 9 yr. We also calculated percentage risk rates to facilitate comparison of our results to those reported by Gurol-Urganci et al [17]. Smaller studies revealed similar results [18], [19], [20].

However, those reoperation rates cannot be considered an accurate evaluation of long-term mesh-related complications for several reasons. First, MUS complications are likely to be underdiagnosed. Over the study period, there was no incentive to provide routine long-term follow-up for MUS patients according to the national guidelines [21] and most surgeons would not see patients again after their first postoperative visit. Hence, only patients complaining of symptoms and seeking care after MUS insertion may ultimately have had a potential complication diagnosed. In this subgroup, it is likely that many women did not undergo an appropriate diagnostic evaluation, as there was no national regulation of SUI management at that time, with no restrictions on what centers and surgeons could perform MUS insertion procedures or subsequent complication management. Therefore, many surgeons and centers had no access to basic diagnostic tool such as urethrocystoscopy and urodynamics.

We found that MUS removal and revision rates were higher for the RP group. Few other studies have compared effectiveness and complication rates between RP and TO groups. A meta-analysis by Leone Roberti Maggiore et al [11] showed similar effectiveness results, with no significant difference in complication rates between the groups. A 2017 Cochrane review [2] of short-term MUS effectiveness that included 36 trials involving 5514 women revealed no significant difference between TO and RP groups. However, Comité d’Urologie et Pelvipérinéologie de la Femme [22] recently demonstrated that each MUS type is associated with its own complications. Intraoperative bladder perforation, suprapubic pain; acute urinary retention, and voiding symptoms are more common with RP slings, while vaginal exposure and groin pain are more common with TO slings. These differences may explain the differing reoperation rates we observed. Bladder outlet obstruction, which is the main issue with RP slings, can easily be managed with sling division or partial excision, which every implanting surgeon is able to perform. Groin pain is much more complex and multifactorial, but most importantly requires full MUS removal, which very few surgeons can perform because of the anatomic challenge of accessing the TO portion of the sling [23].

The proportion of patients who underwent subsequent SUI surgery cannot be regarded as an accurate estimate of the risk of persistent/recurrent SUI after MUS insertion. A significant proportion of patients with immediate or long-term MUS failure may not have sought care because they were disappointed with the outcome of their first procedure. Another hypothesis is that a significant proportion of the subgroup who sought care for recurrent/persistent SUI did not undergo any subsequent procedure as treatment of recurrent SUI remains poorly codified. Thus, our findings may overestimate long-term MUS efficacy.

Advanced age was associated with lower risk of MUS removal and other surgeries for SUI. These results may possibly be explained by a lower benefit/risk ratio for these surgeries in elderly patients.

We observed higher removal and reoperation rates in the obesity and alcohol/tobacco subgroups. There is broad evidence that obesity is an independent risk factor for incontinence [24], [25], [26], which may explain higher reoperation rates for recurrence of incontinence and difficulty in healing in this population. The same risks associated with alcohol and tobacco have been confirmed in other studies [27], [28], [29].

The risk of MUS removal was lower in the outpatient group than in the inpatient group. Patients scheduled for outpatient surgery can easily be hospitalized in the event of pain or postoperative urinary retention. Thus, outpatient surgery should not be regarded as a predictive factor but more as a surrogate for an experienced surgeon/center.

Our study has several limitations inherent to its retrospective nature and potential information bias when querying by CCAM codes. Surgeons may not always be rigorous when coding; however, the health insurance companies that finance the hospitals apply rigorous controls. We had to choose the right codes to use from the voluminous French data. We excluded codes N394 (Other specified forms of urinary incontinence) as it is not a precise definition of SUI, and JRGA004 (Total removal of a synthetic suburethral sling, by laparoscopy and vaginal approach) because it applies to a minority of patients; however, some patients may have been assigned this code and would thus have been missed.

As part of the SNDS monitoring scheme, it is possible to identify and access patient information for those hospitalized in either private or public health care facilities. Incidents (deaths or complications) that occurred outside of hospital settings were not accessible.

In addition, we were only able to identify coded surgical procedures, which could introduce a possible selection bias. However, we consider that this population is negligible. Concerning the CCAM codes, there has been no recent modification in the typology.

Many data were lacking, especially details for the reoperation technique used, the specialty of the surgeon (gynecologist or urologist), and the center where the procedure was performed. Previous anti-incontinence procedures before the study period could not be captured and an unknown proportion of the study population had previous SUI surgery (eg, more salvage RP than TO procedures, which may explain the higher reoperation rate in this group).

## 5 Conclusions

At 10 yr after initial MUS insertion, we estimated rates of 2.7% for MUS removal, 4.4% for new MUS insertion without removal, and 2.8% for other subsequent SUI surgeries (periurethral balloon, bulking agent, artificial urinary sphincter, or Burch colposuspension). These findings reinforce the valuable MUS role in treatment for female SUI. The study findings may help in patient counseling and for treatment decision-making, but should be clearly put in perspective and warrant further investigation.

  ***Author contributions:*** Margaux Laude had full access to all the data in the study and takes responsibility for the integrity of the data and the accuracy of the data analysis.

  *Study concept and design*: Laude, Bentellis, Bailly.

*Acquisition of data*: Laude, Bentellis, Fabre, Bailly.

*Analysis and interpretation of data*: Laude, Bentellis, Bailly.

*Drafting of the manuscript*: Laude, Bentellis, Peyronnet, Bailly.

*Critical revision of the manuscript for important intellectual content*: Laude, Bentellis, Durand, Tibi, Chauleur, Peyronnet, Bailly.

*Statistical analysis*: Laude, Bentellis, Fabre, Bailly.

*Obtaining funding*: None.

*Administrative, technical, or material support*: Laude, Bentellis, Fabre, Bailly.

*Supervision*: Bentellis, Peyronnet, Bailly.

*Other*: None.

  ***Financial disclosures:*** Margaux Laude certifies that all conflicts of interest, including specific financial interests and relationships and affiliations relevant to the subject matter or materials discussed in the manuscript (eg, employment/affiliation, grants or funding, consultancies, honoraria, stock ownership or options, expert testimony, royalties, or patents filed, received, or pending), are the following: None.

  ***Funding/Support and role of the sponsor:*** None.
