# Supplementary material for: Long-term Survival of Midurethral Mesh Slings for Women with Stress Urinary Incontinence
Source: Eur Urol Open Sci. 2025 Dec 4;83:91–8. doi: 10.1016/j.euros.2025.11.012 (PMC12721317; doi:10.1016/j.euros.2025.11.012)
Supplement: Supplementary Table 1 [file mmc1.docx]

**Supplementary table 1.** CCAM Codes Used in Defining Stress Urinary Incontinence Operations

| **Procedure description** | **CCAM codes** |
| --- | --- |
| ***Mid-urethral sling (MUS) insertions*** |  |
| Introduction of retropubic tape | JDDB007 |
| Introduction of transobturator tape | JDDB005 |
|  |  |
| ***Mid-urethral sling removal procedures*** |  |
| Total removal | JRGA003 |
| Partial removal | JRGA002, JRGC001 |
| Unspecified removal | JRGA001 |
| Section | JRPA001 |
|  |  |
| ***Non-mesh SUI operations*** |  |
| Bulking injection  *Intraurethral submucosal injection of heterologous material, by endoscopy* | JELE001 |
| Infraurethral plication, via vaginal approach | JMBA001 |
| Anterior colpoperineorrhaphy | JLCA007 |
| Posterior colpoperineorrhaphy | JLCA004s |
| Cervicocystopexy by strip via vaginal approach and transcutaneous route, with endoscopic guidance | JDDA003 |
| Direct cervicocystopexy by infraurethral strip, by laparotomy and transvaginally | JDDA006 |
| Direct cervicocystopexy by infra-urethral prosthetic strip, by laparotomy | JDDA005 |
| Indirect cervicocystopexy to the pectineal ligament [of Cooper], by laparotomy | JDDA002 |
| Bladder support by infraurethral myoplasty, via vaginal approach | JMDA001 |
| Release of the urethra [Uretrolysis], by direct approach | JEPA007 |
| Placement of a pericervical urinary sphincter prosthesis in women, by laparotomy | JELA001 |
| Indirect cervicocystopexy via vaginal and abdominal approach, with endoscopic guidance | JDDA008 |
| Meatoplasty or urethral meatotomy without flap, in adults | JEMA017 |

Abbreviations: MUS (Mid-urethral sling); SUI (stress urinary incontinence); CCAM (the *French Common Classification of Medical Acts)*

**Supplementary table 1**.**b** CCAM codes Used to Identify Concomitant Prolapse Operations

| Total hysterectomy, by laparoscopy and vaginal approach | JKFA018 |
| --- | --- |
| Total hysterectomy, by laparotomy | JKFA015 |
| Subtotal hysterectomy, by laparotomy | JKFA024 |
| Total hysterectomy with unilateral or bilateral adnexectomy, by laparotomy | JKFA028 |
| Total hysterectomy with unilateral or bilateral adnexectomy, via vaginal approach | JKFA005 |
| Total hysterectomy with unilateral or bilateral adnexectomy, by laparoscopy and vaginal approach | JKFA006 |
| Total hysterectomy, vaginal approach | JKFA026 |
| Subtotal hysterectomy, by laparoscopy | JKFC002 |
| Total hysterectomy with unilateral or bilateral adnexectomy, by laparoscopy | JKFC003 |
| Total hysterectomy, by laparoscopy | JKFC005 |
| Subtotal hysterectomy with unilateral or bilateral adnexectomy, by laparoscopy | JKFC006 |
| Subtotal hysterectomy with unilateral or bilateral adnexectomy, by laparotomy | JKFA032 |
| Trachelectomy on remaining cervix, via vaginal approach | JKFA011 |
| Trachelectomy on remaining cervix, by laparotomy | JKFA009 |
| Trachelectomy [Cervicectomy] [Amputation of the cervix], via vaginal approach | JKFA008 |
| Total hysterectomy with unilateral or bilateral annexectomy and anterior or posterior colpoperineorrhaphy, via vaginal approach | JKFA021 |
| Total hysterectomy with anterior or posterior colpoperineorrhaphy, via vaginal approach | JKFA025 |
| Total hysterectomy with anterior and posterior colpoperineorrhaphy, via vaginal approach | JKFA002 |
| Total hysterectomy with unilateral or bilateral adnexectomy and anterior and posterior colpoperineorrhaphy, via vaginal approach | JKFA007 |
| Total hysterectomy with posterior suspension of the dome of the vagina, by laparotomy | JKFA013 |
| Subtotal hysterectomy with posterior suspension of the cervix [colposuspension], by laparotomy | JKFA014 |
| Subtotal hysterectomy with unilateral or bilateral adnexectomy and posterior suspension of the cervix [colposuspension], by laparotomy | JKFA001 |
| Total hysterectomy with unilateral or bilateral adnexectomy and posterior suspension of the dome of the vagina, by laparotomy | JKFA004 |
| Subtotal hysterectomy with posterior suspension of the cervix [colposuspension] and indirect cervicocystopexy to the pectineal ligament [of Cooper], by laparotomy | JKFA012 |
| Subtotal hysterectomy with unilateral or bilateral adnexectomy, posterior suspension of the cervix [colposuspension] and indirect cervicocystopexy to the pectineal ligament [of Cooper], by laparotomy | JKFA029 |
