# Supplementary material for: Long-term Survival of Midurethral Mesh Slings for Women with Stress Urinary Incontinence
Source: Eur Urol Open Sci. 2025 Dec 4;83:91–8. doi: 10.1016/j.euros.2025.11.012 (PMC12721317; doi:10.1016/j.euros.2025.11.012)
Supplement: Supplementary Table 2 [file mmc2.docx]

Table 2. Population characteristics and post operative complications to identify potential predictive factors

| **Population characteristics** | **Diagnostic codes of the 10th International Classification of Diseases (ICD10)** |
| --- | --- |
| **Age of patient at admission** |  |
| **Diabetes** | E10 to E14 |
| **Obesity** | E66 |
| **Cardiovascular diseases:**  - Dyslipidemia  - Arterial hypertension:  - Congestive heart failure  - Ischemic heart disease  - Other forms of heart disease:  *left bundle branch and atrioventricular block*  *other conduction disorders*  *paroxysmal tachycardia*  *atrial fibrillation and flutter*  *other cardiac arrhythmias*  *heart failure*  - Carotid artery stenosis, cerebral atherosclerosis  - Atherosclerosis of other arteries (unspecified) | E78  I10-I13, I15  I09.9, I11.0, I13.0, I13.2, I25.5, I42.0, I42.5 to I42.9, I43, I50, P29.0  I20, I21, I22, I24, I25  *I44*  *I45*  *I47*  *I48*  *I49*  *I50*  I65.2, I67.2  I70.8 (I70.80, I70.81), I70.9 (I70.90, I70.91) |
| **Neurological diseases:**  - Neurological disorders  - History of stroke  - Hemiplegia  - Dementia | G138, G64, G909, G31, G968, G978, G979, G90, G99, P11, P14, O743  I61, I63, I64, G45  G81  F01, F03 |
| **Cancers:**  - Leukemia  - Lymphoma  - Cancer | C90 to C95  C81 to C86  C00 - C26, C30 - C34, C37 - C41, C45 - C58, C60 - C76, C77-C80, C97 |
| **Alcohol, smoking and drug disorders:**  - Smoking  - History of alcoholism  - History of drug use | F17  F10, E52, G62.1, I42.6, K29.2, 70.0, K70.3, K70.9, T51, Z50.2, Z71.4, Z72.1  F11-F16, F18, F19, Z71.5, Z72.2 |
| **Chronic illnesses other than diabetes:**  - Chronic respiratory tract diseases  - Connectivity  - History of digestive ulcers  - Chronic liver disease  - Chronic renal failure (stage 4 or 5 or dialysis) | J41, J42, J44-J47, J96.1, J96.99  M30 to M36  K22, K25, K26, K27, K28  B18, I85, I86.4, I98.2, K70, K71.1, K71.3 - K71.5, K71.7, K72-K74, K76.0, K76.2 - K76.9, Z94.4  N18.4, N18.5, Z99.2 |
| **Anemia and Hemostasis Disorders:**  - History of major bleeding  - Coagulopathy  - Anemia | I60-I62, D62, J94.2, H11.3, H31.3, H35.6, H43.1, N02, N95, R04, R31, R58, K25.0, K25. 2, K25.4, K25.6, K26.0, K26.2, K26.4, K26.6, K27.0, K27.2, K27.4, K27.6, K28.0, K28.2, K28.4, K28.6, K29.0, K62.5, K92.0-K92.2, S06.3-S06.6  D65-D68, D69.1, D69.3-D69.6  D50.0, D50.8, D50.9, D51-D53 |
| **Pelvic static disorder and other pelvic malformations:**  - Genital prolapse  - Congenital uterovaginal and urinary tract malformations | N81, K62, N993, Q518, O655-8  Q51, Q52, Q628, Q649 |
| **Post operative complications** |  |
| **Bleeding:**  -Hemorrhage, hematoma  -Transfusions of blood products | T81.0 to T81.2  Z51-3 and Z51-30 |
| **Site infections:**  -Disunion of a wound  -Surgical site infection | T81.3 and T81.38  T81.4 and T81.8 and T81.9 |
| **Bladder or vaginal wounds:**  -Bladder perforation  -Bladder disorder unspecified  -Genital fistulas | S372  N329  N82.0 to N82.9 |
| **Complications of genitourinary prostheses:** | T83, T856, T857, T858, T859, T868, Y831, Z969, Z470, Z45, Z466, T888, T889, T813 |
| **Symptomatic emptying disorders**  -Dysuria:  -Neurological bladder:  -Urinary tract infection, distant from installation: | R300  N31.0 to N31.2  N390 |
